# Randomized comparison of tape versus semi-rigid and versus lace-up ankle support in the treatment of acute lateral ankle ligament injury

**DOI:** 10.1007/s00167-015-3664-y

**Published:** 2015-06-05

**Authors:** M. P. J. van den Bekerom, Robert van Kimmenade, I. N. Sierevelt, Karin Eggink, G. M. M. J. Kerkhoffs, C. N. van Dijk, E. E. J. Raven

**Affiliations:** Department of Orthopaedic Surgery and Traumatology, Onze Lieve Vrouwe Gasthuis, Oosterpark 9, P.O. Box 95500, 1090 HM Amsterdam, The Netherlands; Department of Orthopaedic Surgery and Traumatology, Gelre Hospitals, Albert Schweitzerlaan 31, P.O. Box 9014, 7300 DS Apeldoorn, The Netherlands; Orthopaedic Research Center Amsterdam, Department of Orthopaedic Surgery, Academic Medical Centre/University of Amsterdam, Meibergdreef 15, P.O. Box 22660, 1105 AZ Amsterdam, The Netherlands; Department of Orthopaedic Surgery and Traumatology, Slotervaart Hospital, Louwesweg 6, P.O. Box 90440, 1006 BK Amsterdam, The Netherlands

**Keywords:** Ankle, Ligament, Sports, Injury, Treatment, Brace, Tape

## Abstract

**Purpose:**

Functional treatment is the optimal non-surgical treatment for acute lateral ankle ligament injury (ALALI) in favour of immobilization treatment. There is no single most effective functional treatment (tape, semi-rigid brace or lace-up brace) based on currently available randomized trials.

**Methods:**

This study is designed as a randomized controlled trial to evaluate the difference in functional outcome after treatment with tape versus semi-rigid versus lace-up ankle support (brace) for grades II and III ALALIs. The Karlsson score and the FAOS were evaluated at 6-month follow-up.

**Results:**

One hundred and ninety-three patients (52 % males) were randomized, 66 patients were treated with tape, 58 patients with a semi-rigid brace and 62 patients with a lace-up brace. There were no significant differences in any baseline characteristics between the three groups. Mean age of the patients was 37.3 years (35.1–39.5; SD 15.3). Ninety-five males (49 %) were included. One hundred and sixty-one (59 + 50 + 52) patients completed the study through final follow-up; 32 % lost at follow-up. In two patients treated with tape support, the treatment was changed to a semi-rigid brace because of dermatomal blisters. Except for the difference in Foot and Ankle Outcome Score sport between the lace-up and the semi-rigid brace, there are no differences in any of the outcomes after 6-month follow-up.

**Conclusion:**

The most important finding of current study was that there is no difference in outcome 6 months after treatment with tape, semi-rigid brace and a lace-up brace.

**Level of evidence:**

I.

## Introduction

Each year, approximately 520,000 persons in the Netherlands (16 million inhabitants) sustain a lateral ankle ligament injury, of which about 200,000 are a result of sports [8]. About half of the patients with these injuries receive medical treatment, and 40 % of injuries result in chronic symptoms [2, 25]. Of the patients who play sports, around 60–90 % resumed sports after 12 weeks at the same level as before the trauma [18]. Especially basketball, football, soccer and volleyball have a high incidence of ankle injuries [11, 24, 25].

The lateral ankle ligament complex consists of the anterior talofibular, the calcaneal fibular and the posterior talofibular ligaments (respectively, ATFL, CFL and PTFL) [18]. However, the number of ligaments injured does not affect the prognosis and is therefore not considered relevant for the treatment [3]. The most common mechanism of injury is supination and adduction (usually referred to as inversion) of the plantar-flexed foot. Ankle inversion injuries are usually classified according to a three-stage grading system: grade I is a mild stretching of the ligament without instability of the joint, grade II is a partial rupture of the ligament with mild instability of the joint and grade III involves complete rupture of the ligaments with instability of the joint [3, 21]. Due to pain and swelling, classification of patients is often only possible after 4–5 days [22].

The three main modalities of treatment for inversion injuries are as follows: (1) surgical treatment, (2) conservative treatment by immobilization with a plaster cast or splint and (3) functional conservative treatment with tape, semi-rigid brace or lace-up brace.

A Cochrane systematic review (20 RCTs, *n* = 2562) of surgical versus conservative treatment for acute ankle ligament injuries was inconclusive (due to insufficient evidence) with regard to the superiority of surgical treatment [5]. It is recommended to consider surgical treatment for (top professional) sports players on an individual basis [8, 12, 17].

A second Cochrane systematic review (21 RCTs, *n* = 2184) showed that functional treatment is superior to immobilization as conservative treatment for patients with acute lateral ankle ligament injuries [6].

According to the Cochrane systematic review (9 RCTs, *n* = 892) concerning different functional treatment options (tape, semi-rigid brace, lace-up brace) for acute ankle ligament injuries, ‘there is no most effective treatment either clinically and in costs based on currently available randomised trials’ [7]. High quality, sufficiently powered randomized trials are warranted to compare the effectiveness of different functional strategies for treatment of an acute ankle sprain [7]. The objective of this study is to determine the foremost optimal functional treatment by comparing tape versus semi-rigid support versus lace-up brace treatment for acute lateral ankle ligament injuries with regard to clinical outcome. Our hypothesis was that (lace-up and semi-rigid) bracing was superior to taping in the treatment of acute lateral ankle ligament injuries.

## Materials and methods

Of all patients with an ankle inversion injury presenting at the emergency department of our hospital, the injury mechanism and general history were acquired. The lower extremity was examined. The presence or absence of an ankle fracture was initially assessed according to the Ottawa ankle rules [15]. If a fracture could not be excluded, radiographs (mortise and lateral) of the ankle were made. When the diagnosis ‘acute ankle sprain’ was made, RICE therapy (Rest, Ice application, Compression with a pressure bandage and Elevation) [20] was started and patients were advised not to bear weight on the injured leg and to walk with crutches until the first visit. During this first visit, which was between 4 and 7 days after the initial trauma, a delayed physical examination was performed. The physical examination was performed as described by Van Dijk [21, 22]. The most important items to assess were pain on palpation, haematoma, and instability evaluated with the anterior drawer test. Patients qualifying for grade II or III ligament injury at this delayed physical examination [22] were eligible and asked to participate in the study. The inclusion and exclusion criteria are summarized in Table 1. Patient demographic characteristics (gender, age, height, weight, dominant leg and occupation) and the pre-injury Tegner activity level are recorded [16].

**Table 1:** Study population—inclusion and exclusion criteria

Inclusion criteria
Age ≥ 18 years
Grades II or III ankle sprains
Presentation on the ER < 72 h after the acute injury
Exclusion criteria
History of chronic instability (instability complaints for more than 6 months)
Fracture on conventional radiograph
Other injuries or disabilities on the same limb
Alcoholism, serious psychiatric and neurological illness
Bilaterally sprained ankles
Previous surgery on the lateral ankle ligaments
Skin diseases where taping is contraindicated
Inability to give informed consent
Inability to fill out questionnaires
Neuromuscular disorders of the lower extremities
Active rheumatoid arthritis
Gait disturbances

### Randomization

This study was designed as a randomized controlled trial to evaluate the difference in functional outcome after treatment with tape versus semi-rigid versus lace-up ankle support (brace) for grades II and III acute lateral ankle ligament injuries. Randomization was performed using online randomization software.

### Interventions

Use and application were explained by the researcher using a standardized protocol. In case of complications, another required treatment was started, but the patient will be evaluated according to the intention-to-treat principle. Apart from the investigated treatment, patients underwent the same rehabilitation programme: active range of motion training, weight bearing as tolerated, and use of crutches until the pain subsides and full weight bearing is reached. The use of additional treatment (ultrasound, cryotherapy, laser, homeopathy and physiotherapy) was not allowed. Analgesics were allowed, and although nowadays there is evidence to support the use of non-steroidal anti-inflammatory drugs (NSAIDs) [19], these and morphine–mimetic drugs were not allowed. After 6 weeks, the treatment with tape or (semi-rigid/lace-up) ankle support was terminated.

#### Follow-up

After the initial visit (4–7 days after the initial trauma) and inclusion in the study, patients were assessed at 2 weeks, 4 weeks and 6 months. At 2 and 4 weeks, the outcome was not assessed; only complications were registered, and the compliance with the treatment was optimized. At 6 months, the final outcome was assessed.

### Outcome assessment

At 6 months, relevant outcome data were collected through clinical evaluation performed by the orthopaedic surgeon or resident in orthopaedic surgery. As blinding of patients was not possible, the resident or surgeon was blinded. At the final assessment, the patients were instructed not to talk about the type of treatment with the outcome assessor.

1. Karlsson scoring scale [4]. This score is widely used to assess ankle function after ligament injury [1]. The patients are asked to fill out a questionnaire regarding the function of the ankle joint. The score includes eight items based on a subjective evaluation of stability, pain, swelling and stiffness in relation to activities of everyday life, sports and recreational activities, running, stair climbing and working ability. The minimum is score is 0, and the maximum is 100. There is no reliable information available concerning the reliability, the internal consistency, the test–retest reliability and the time needed to fill out the questionnaire [4].
2. Foot and Ankle Outcome Score (FAOS) [13, 14]. The FAOS consists of five subscales: pain, other symptoms, function in daily living (ADL), function in sport and recreation (Sport Rec) and foot- and ankle-related quality of life (QOL). The last week is taken into consideration when answering the questionnaire. Standardized answer options are given (% Likert boxes), and each question gets a score from 0 to 4. A normalized score (100 indicating no symptoms and 0 indicating extreme symptoms) is calculated for each subscale. The result can be plotted as an outcome profile. FAOS content is based on the Knee injury and Osteoarthritis Outcome Score (KOOS) [13], and content validity was confirmed by 213 patients with ankle instability [14].
3. Return to work (time to return to work).
4. Return to sports at the same level as pre-injury (time to return to sports).
5. Pain at rest VAS score (0 = no pain, 10 = unbearable pain).
6. Brace- or tape-related complications or adverse events (yes/no).
7. Tegner activity level [16].
  The Tegner activity scale is designed as a score of activity level to complement other functional scores (e.g. the Lysholm knee score) for patients with ligamentous injuries.

### Institutional review board (IRB)

Ethical approval (with Document No. NL27757.041.09, ABR No. 27757 and METC No. 09-142) was obtained at the ethical committee of the University of Utrecht. This trial was registered (NCT01126242) (date of registration and date of enrolment of first patient: 18 May 2010).

### Sample size

The data of Boyce et al. [1] were used to estimate the required sample size of the study. To protect against an inflated type-I error (due to three pairwise comparisons), an alpha level of 0.017 was used for each comparison with respect to the control group (Bonferroni correction). We hypothesized that there was a difference of 10 in functional outcome (Karlsson Score [1]) between non-elastic adhesive taping and semi-rigid and lace-up ankle support, in favour of the last, for the treatment of acute lateral ankle ligament injury at 6-month follow-up. Using a power of 80 %, a difference of 10 in Karlsson score at 8 weeks, and a standard deviation of 15, it was estimated that 56 patients per group were required. It was assumed that 10 % of the patients would drop out and, consequently, we aimed to include 62 patients per group.

### Statistical analysis

Unique case report forms (CRF) were used to collect the data obtained during this study. Analysis of the data was performed anonymously by use of SPSS version 21 (IBM, Chicago, IL, USA). Continuous data were checked for normality (Kolmogorov–Smirnov test) and described as means with SD in case of normal distribution, otherwise as medians with ranges. Categorical data are presented as numbers with percentages.

The patient’s baseline characteristics are described and compared between groups. Continuous data were analysed using ANOVA tests. Chi-squared tests were performed in case of dichotomous variables.

Statistical analysis of the outcome measures was performed according to the intention-to-treat principle. For each outcome measure, the change from baseline at 6 months was calculated and presented as mean with 95 % CI. Comparisons between the treatment groups were made by use of a one-way analysis of variance (ANOVA). A *p* value <0.05 was considered statistically significant. In case of significance, post hoc pairwise comparisons (Student’s *t* tests) were performed with adjusted significance levels for multiple testing (Bonferroni, *p* < 0.017). Differences of categorical scales (return to work/sport) between the treatment groups were analysed by use of a Chi-square test (significance level, *p* < 0.05).

## Results

One hundred and ninety-three patients (52 % males) were randomized, 66 patients were treated with tape, 58 patients with a semi-rigid brace and 62 patients with a lace-up brace. The number of patients eligible to be enrolled in the study but who declined, and the reasons therefore, is not known. Table 2 shows the baseline characteristics of all subjects enrolled in the study. There were no significant differences in any baseline characteristic between the three groups. Mean age of the patients was 37.3 years (35.1–39.5; SD 15.3); the youngest patient was 18, and the oldest was 78. Ninety-five males (49 %) were included. In 48 % of patients, the right side was injured, and in 52 % of patients, the left side was injured. In 50 % of the patients, the dominant side was injured, and in the other 50 %, the non-dominant side was injured. Fifty-one per cent of the patients had a Tegner score of 1, 24 % had a score of 2 and 25 % had a higher Tegner score. Concerning the anterior drawer test, 11 % had a negative test, 55 % had a 1 + test, 32 % had a 2 + test and no patients had a 3 + test. Thirty-nine per cent of the patients had a haematoma at the lateral side of the ankle, and 39 % of the patients had diffuse swelling of their ankle. One hundred and sixty-one (59 + 50 + 52) patients completed the study through final follow-up. In two patients treated with tape support, the treatment was changed to a semi-rigid brace because of dermatomal blisters. Except for the difference in FAOS sport between the lace-up and the semi-rigid braces, there are no differences in any of the outcome measures after 6-month follow-up (Table 3).

**Table 2:** Baseline characteristics of included patients (mean with SD)

	Tape	Semi-rigid brace	Lace-up brace	*p* value
Patients included	66	58	62	
Mean age (years) (range)	35 (15)	40 (16)	37 (15)	n.s.
Gender (male/female)	33 (50 %)/33 (50 %)	32 (55 %)/26 (45 %)	30 (48 %)/32 (52 %)	n.s.
Injured side (left/right)	39 (59 %)/27 (41 %)	27 (47 %)/31 (53 %)	33 (53 %)/29 (27 %)	n.s.
Haematoma	42 (63 %)	32 (56 %)	40 (64 %)	
Swelling	40 (61 %)	32 (56 %)	42 (68 %)	
Karlsson score	37 (27)	40 (27)	35 (28)	n.s.
VAS pain	48 (23)	53 (25)	52 (24)	n.s.
FAOS				
Symptoms	53 (18)	58 (20)	53 (18)	n.s.
Pain	50 (21)	52 (20)	48 (20)	n.s.
ADL	53 (21)	52 (22)	50 (20)	n.s.
Sport	38 (38)	41 (36)	33 (31)	n.s.
QOL	41 (16)	43 (16)	41 (17)	n.s.
Tegner score	1 (2)	1 (3)	2 (2)	n.s.
VAS health	71 (20)	72 (23)	69 (22)	n.s.

**Table 3:** Outcome assessment at 6 months, change from baseline (mean with 95 % CI)

	Tape	Semi-rigid brace	Lace-up brace	*p* value
Patients assessed (*n*, %)	59 (89 %)	50 (86 %)	52 (84 %)	0.66
Karlsson score	32 (22–42)	33 (22–43)	40 (30–50)	0.47
VAS pain	−24 (−15 to −85)	−33 (−42 to −24)	−33 (−42 to −24)	0.21
FAOS				
Symptoms	24 (16–31)	20 (14–26)	27 (21–36)	0.33
Pain	27 (19–34)	28.9 (22–36)	38 (31–45)	0.07
ADL	27 (19–35)	30 (22–38)	40 (28–37)	0.07
Sport	33 (21–46)	25 (14–37)*	49 (39–60)	0.02
QOL	15 (10–21)	15 (9–21)	20 (14–26)	0.34
Tegner score	3 (2–4)	2 (1–3)	2 (2–3)	0.53
VAS health	0.4 (−6 to 7)	10 (3–17)	7 (−1 to 15)	0.17
Return to work				
No return	22 % (12/55)	10 % (4/41)	9 % (4/44)	0.30
Below level	15 % (8/55)	12 % (5/41)	11 % (5/44)	
Normal level	63 % (35/55)	78 % (32/41)	80 % (35/44)	
Return to sport				
No return	5 % (2/39)	11 % (4/35)	8 % (3/39)	0.65
Below level	33 % (13/39)	37 % (13/35)	26 % (10/39)	
Normal level	62 % (24/39)	52 % (18/35)	66 % (26/39)	

* Sign difference between semi-rigid and lace-up braces (*p* = 0.003)

## Discussion

The most important finding of current study is that there is no difference in outcome between tape, semi-rigid brace and a lace-up brace 6 months after treatment. Functional treatment is favoured over immobilization treatment and is considered the optimal non-surgical treatment for acute lateral ankle ligament injury [6]. The purpose of this study is to determine whether tape, brace or lace-up brace treatment is the optimal functional non-surgical treatment for acute lateral ankle ligament injury. In all treatment groups, patients scored better functional results (Karlsson and FAOS) compared to baseline at follow-up, but there were no difference between the groups at 6-month follow-up. These conclusions are similar to those drawn in the Cochrane review [6]. The patients were followed for 6 months because the most recovery occurs within the first 6 months after the injury, and re-injury rates stabilize thereafter [23]. Most ankle sprain patients (75–100 %) have a 1-year outcome that is excellent or good and fully acceptable to the patients irrespective of the therapy they received. Therefore, achieving optimalization of treatment is relevant especially for the short and intermediate terms [3].

Although economic benefits of the treatment options are not an outcome in our study, the cost of brace treatment is presumably lower than the cost of tape treatment. The costs of the brace itself are higher, but the patient can apply the brace himself after the initial period immediately following the trauma. Patients treated with tape need to visit the hospital and general practitioners or physiotherapist need to reapply the tape every 2 weeks. Lardenoye and colleagues show that treatment of acute lateral ankle ligament injury with a semi-rigid brace leads to fewer complications, higher patient satisfaction and similar functional outcome compared to treatment with tape [10]. Based on our trial and currently available evidence [6, 7, 9, 10], applying a below-knee cast for a short period of time and subsequent use of a semi-rigid or lace-up brace for a period of up to 6 weeks is recommended as treatment for a grades II or III lateral ankle ligament injury.

When this study was designed, great effort was made to minimize potential sources of confounding and bias. The strength of current randomized trial is that it was initiated after a proper sample size calculation. The outcome assessor was blinded. In contrast to other trials, grade 1 ankle sprains were not included because these injuries do not need a supportive treatment.

A limitation of current study is that the treatment providers and study participants were not blinded to assignment status after allocation because we were unable to conceal the specific external support the subject was wearing. Comparisons between the findings from this study and those of prior reports in the literature and other populations in society need to be done carefully, considering differences in the patient populations studied. An ankle sprain is considered a typical sport injury, but the Tegner score was only 1 or 2 in 75 % of patients included in this study. Another limitation is that it was not possible to measure compliance with the supports accurately.

Form previous research, there is known that the majority of the patients can be treated with a functional type of treatment. From current research, we know that there is no difference in outcome between these types of functional treatments and therefore is all an option to treat patients with an acute lateral ankle ligament injury in clinical practice. A potential advantage of the braces (semi-rigid and lace-up) may be that these braces can be given to the patients when they visit the emergency department. Patients can apply this brace themselves 4–7 days after trauma while patients who are treated with tape have to see somebody who can apply this tape. This potential advantage cannot be concluded from current trial because all patients were evaluated at the same time points and all patients were evaluated during these follow-up visits.

A future trial should be a large multicentre trial with patient reported outcome measures, patient satisfaction analysis and a cost-effectiveness analysis. The treatment options should be compared after an initial short period of immobilization. After the Cochrane review [7] and the unpublished update of this review including this trial, no major functional differences can be expected between the different types of functional treatment.

## Conclusion

The most important finding of current study was that there is no difference in outcome 6 months after treatment of acute lateral ankle ligament injury with a tape, semi-rigid brace and a lace-up brace.
